# Physical shearing imparts biological activity to DNA and ability to transmit itself horizontally across species and kingdom boundaries

**DOI:** 10.1186/s12867-017-0098-8

**Published:** 2017-08-09

**Authors:** Gorantla Venkata Raghuram, Deepika Gupta, Siddharth Subramaniam, Ashwini Gaikwad, Naveen Kumar Khare, Malcolm Nobre, Naveen Kumar Nair, Indraneel Mittra

**Affiliations:** Translational Research Laboratory, Advanced Centre for Treatment, Research and Education in Cancer, Tata Memorial Centre, Navi-Mumbai, 410210 India

**Keywords:** Sonicated DNA, DNA fragments, Indiscriminant DNA uptake by cells, Nuclear uptake of foreign DNA, Genomic integration of foreign DNA, Biological activation of DNA, Mobile genetic elements, Horizontal gene transfer, Evolution of species

## Abstract

**Background:**

We have recently reported that cell-free DNA (cfDNA) fragments derived from dying cells that circulate in blood are biologically active molecules and can readily enter into healthy cells to activate DNA damage and apoptotic responses in the recipients. However, DNA is not conventionally known to spontaneously enter into cells or to have any intrinsic biological activity. We hypothesized that cellular entry and acquisition of biological properties are functions of the size of DNA.

**Results:**

To test this hypothesis, we generated small DNA fragments by sonicating high molecular weight DNA (HMW DNA) to mimic circulating cfDNA. Sonication of HMW DNA isolated from cancerous and non-cancerous human cells, bacteria and plant generated fragments 300–3000 bp in size which are similar to that reported for circulating cfDNA. We show here that while HMW DNAs were incapable of entering into cells, sonicated DNA (sDNA) from different sources could do so indiscriminately without heed to species or kingdom boundaries. Thus, sDNA from human cells and those from bacteria and plant could enter into nuclei of mouse cells and sDNA from human, bacterial and plant sources could spontaneously enter into bacteria. The intracellular sDNA associated themselves with host cell chromosomes and integrated into their genomes. Furthermore, sDNA, but not HMW DNA, from all four sources could phosphorylate H2AX and activate the pro-inflammatory transcription factor NFκB in mouse cells, indicating that sDNAs had acquired biological activities.

**Conclusions:**

Our results show that small fragments of DNA from different sources can indiscriminately enter into other cells across species and kingdom boundaries to integrate into their genomes and activate biological processes. This raises the possibility that fragmented DNA that are generated following organismal cell-death may have evolutionary implications by acting as mobile genetic elements that are involved in horizontal gene transfer.

**Electronic supplementary material:**

The online version of this article (doi:10.1186/s12867-017-0098-8) contains supplementary material, which is available to authorized users.

## Background

Several hundred billion to a trillion cells are known to die in the adult human body daily and a considerable amount of resulting fragmented DNA and/or nucleosomes enter the blood stream [1, 2]. These DNA and/or nucleosome fragments have been variously termed as circulating nucleic acids (CNAs), cell-free nucleic acids (cfNAs), cell-free DNA (cfDNA), DNA fragments (DNAfs) and chromatin fragments (Cfs) [3–6].

It is well known that bacteria can take up extraneous DNA [7]. However, introduction of DNA into mammalian cells require permeabilization of the cell membrane [8]. On the other hand, several reports suggest that apoptotic DNA can be spontaneously taken up by mammalian cells. Bergsmedh et al. [9] showed transfer of oncogenes following co-culture of apoptotic bodies with mouse fibroblast cells which led to their oncogenic transformation [9]. DNA released from leukemic cells can get integrated into chromosomes of surrounding stromal cells which result in DNA damage and apoptosis [10]. Mutated *K*-*ras* oncogene present in plasma of colon cancer patients were shown to be taken up by mouse fibroblast cells, leading to their cancerous transformation; and *K*-*ras* sequences could be detected in them by PCR and FISH [11, 12]. Reconstitution of genes in vitro into chromatin were reported to be readily taken up by cells in culture to localize in the nuclei of recipient cells, which led to the suggestion that chromatinized DNA maybe an efficient means for gene delivery [13]. The detection of male foetal DNA in maternal brain cells suggests that circulating foetal DNA can cross the blood–brain barrier [14]. We have recently reported that fragmented cfDNA that circulate in human blood are not only readily taken up by mammalian cells but that they can evoke biological responses in the recipients [3, 4, 6, 15]. When DNA was isolated from blood of cancer patients and healthy volunteers and added to cells in culture, they were rapidly taken up by the recipient cells to accumulate in their nuclei [3, 4, 6]. The internalized DNA associated themselves with host cell chromosomes triggering a DNA damage repair response which facilitated their genomic integration [4]. Damage to DNA also activated an apoptotic response resulting in death of some cells [4].Taken together, the above studies support the observation that fragmented cfDNAs can be internalized by healthy cells with potentially oncogenic consequences.

In order to explain the above findings, we hypothesized that cellular entry and acquisition of biological activity are functions of the size of DNA. We show that while HMW DNAs were incapable of entering into cells, sonicated DNA (sDNA) from various sources were taken up by each other indiscriminately without heed to species or kingdom boundaries. The internalized sDNAs associated themselves with host cell chromosomes and integrated into their genomes. They also evinced biological activities in the form of their ability to phosphorylate H2AX and activate the pro-inflammatory cytokine NFκB. Our results raise the possibility that fragmented DNA from dying cells may act as mobile genetic elements with evolutionary implications by being involved in horizontal gene transfers. Acute environmental stress leading to organismal cell-death and DNA fragmentation may have played an important role in evolutionary processes.

## Methods

### Sources of DNA

HMW human DNA was isolated from WI-38 human normal lung fibroblasts and MDA-MB-231 human breast carcinoma cells. Both cell-lines were obtained from American Type Culture Collection: catalogue numbers ATCC^®^ CCL-75™ and ATCC^®^ HTB-26™, respectively, and grown in Dulbecco’s Modified Eagle’s Medium containing 10% foetal bovine serum. Bacterial DNA was isolated from *Escherichia coli* DH5α (a gift from Dr. Kakoli Bose) grown in Luria Broth (Hi-Media, Mumbai. India. Cat. No. M575). Plant DNA was isolated from *Musa acuminata* (banana) leaves obtained from plants growing wildly in our institute garden.

### Isolation of HMW and sonicated DNA

HMW DNA was isolated from WI-38 and MDA-MB-231 cells using DNeasy Blood & Tissue Kit (Catalogue No. 69506; Qiagen, Limburg, Netherlands) according to manufacturer’s protocol. Bacterial DNA was isolated from an overnight culture of *E. coli* DH5α using Wizard^®^ Genomic DNA Purification Kit (Catalogue No. A1125; Promega Corporation, Madison, WI, USA) as per manufacturer’s protocol. Plant DNA was isolated from banana leaves using the HiPurA™ Plant Genomic DNA Miniprep Purification Kit (Catalogue No. MB507; HIMEDIA Laboratories Pvt. Ltd., Mumbai, India). Leaves were cut into small pieces and were ground with liquid nitrogen using a mortar and pestle pre-chilled to −20 °C. The yield of DNA from the four different sources was as follows: WI-38: 16.30 µg from 10^6^ cells; MDA-MB-231: 18 µg from 10^6^ cells; bacteria: 57 µg from 1 ml of overnight culture (OD = 0.8); plant: 30.6 µg from 40 mg banana leaf.

HMW DNA (500 ng) in 50 µl of Tris–EDTA buffer, pH 7.5 from all four sources were sonicated at 10% amplitude (64 μ or 0.0025 in.) for 10 s using a double step 1/8″ microtip with coupler and a sonicator (Branson Digital Sonifier^®^ 250/450). This amplitude and timing of sonication was arrived at by hit-and-trial method until we got fragment sizes which were similar to those obtained from sera of human subjects [4]. The HMW and sDNA (500 ng each) were characterized by running on a 1% agarose gel at 100 V for 60 min. A 1 kb DNA ladder (Gene Ruler; Thermo Scientific; Cat. No. SM0311) was also run to ascertain the size range of sDNA.

### Flourescent labeling of DNA and confocal microscopy

#### Fluorescent labeling

In order to fluorescently label DNA, WI-38 and MDA-MB-231 cells were grown in DMEM medium containing BrdU (Sigma Chemicals USA, Catalogue No. B5002-100MG) at a final concentration of 10 μM for 24 h. Cells were washed ×3 with PBS, and the labeled HMW and sDNAs were isolated as described above. Bacteria, grown in Luria Broth were labeled with BrdU for 30 min at the above concentration of BrdU and labeled HMW and sDNA were extracted. Thirty minutes of labeling produced satisfactorily observable fluorescent signals under confocal microscope. Unlabeled DNA was not quantified. Since we did not grow plant cells in culture, DNA from plant was non-enzymatically labeled in vitro after HMW DNA extraction. We used Platinum Bright™ 550 Red/Orange Nucleic Acid Labelling Kit (Kreatech Diagnostics, The Netherlands. Catalogue No. GLK-004) in 50 μl reactions. Labelling was done as per manufacturer’s protocol followed by sonication.

#### Treatment of recipient NIH3T3 cells and laser scanning confocal microscopy (LSCM)

NIH3T3 mouse fibroblast cells grown in 35 mm^3^ dishes on cover slips (10 × 10^4^ cells) were treated with fluorescently-labeled HMW and sDNA (10 ng each) isolated from WI-38, MDA-MB-231, bacterial and plant sources. After 30 min of treatment, cells were washed ×3 with PBS and, with exception of cells treated with plant DNA (see below), were processed for LSCM using anti-BrdU antibody (1:100 dilution) (Abcam, USA, Catalogue No. ab6326) and FITC-labeled secondary antibody (1:500 dilution) (Abcam, USA, Catalogue No. ab102263), mounted onto clean glass slides with Vecta-shield and were visualized using Zeiss differential LSCM platform. Since plant DNA was non-enzymatically labeled in vitro, the treated cells were directly visualized under LSCM platform. Fifty nuclei were randomly chosen for analysis in each case and the mean nuclear fluorescence intensity was measured using LSM Image Examiner 4.0 software (Carl Zeiss Jena GmbH, Germany).

#### Treatment of recipient bacterial cells and fluorescent microscopy

Fluorescently-labeled MDA-MB-231, WI-38, bacterial and plant DNA (10 ng each) were added to 2 ml of bacterial culture, incubated for 30 min, processed as above and examined under a fluorescent microscope. One hundred bacteria were analyzed and the number of cells showing positive signals was determined.

### Chromosomal association and genomic integration

NIH3T3 cells were treated with fluorescently labeled HMW and sDNA (10 ng DNA each) as described above and metaphase spreads were prepared after 6 h of treatment and observed under fluorescent microscope. Fifty metaphases were analyzed and the average number of signals per metaphase was recorded.

To investigate possible genomic integration of human DNA (WI-38 and MDA-MB-231), 10 ng DNA each of HMW and sDNA were added to NIH3T3 cells and metaphase spreads were prepared at 10th passage. FISH was performed using human whole genomic and pan-centromeric probes as described by us earlier [4]. The human probes do not cross-react with mouse DNA. Fifty metaphases were analyzed for detection of human DNA signals.

### Detection of γ-H2AX and NFκB by immuno-fluorescence

NIH3T3 cells were treated with HMW and sDNA from all four sources for 6 h and processed for detection of γ-H2AX signals by indirect immuno-fluorescence as described by us earlier [4]. Control cells were treated with 100 μl PBS. Experiments were done in duplicate and 100 DAPI-stained nuclei were randomly analyzed in each case. The average percentage of cells showing positive signals was determined.

NFκB was analyzed by indirect immuno-fluorescence using specific antibodies against NFκB (Abcam^®^, UK. Catalogue No. ab16502). Anti-rabbit secondary antibodies labeled with FITC (Abcam UK. Catalogue Nos. ab6717/ab6785/ab7121) were used. Mean nuclear fluorescent intensity (MFI) was determined in duplicate in 50 cells in each case. Background MFI of untreated cells was deducted from that in treated cells and results were depicted as mean ± S.E.

## Results

**Fig. 1:**
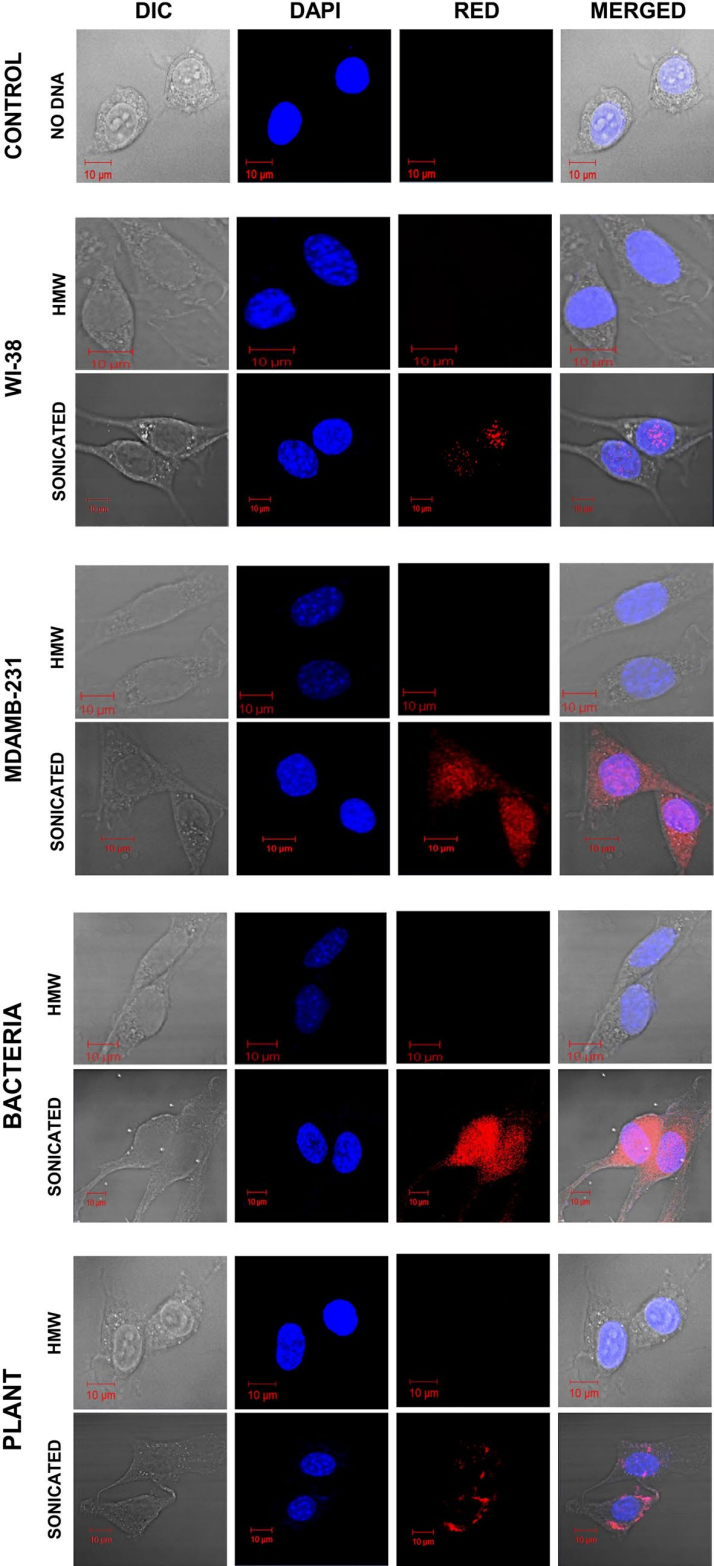
Cellular entry and nuclear uptake of fluorescently labeled HMW and sDNA from various sources by NIH3T3 cells. Images were taken at 30 min following treatment. Representative LSCM images: HMW DNA and sonicated DNA

**Fig. 2:**
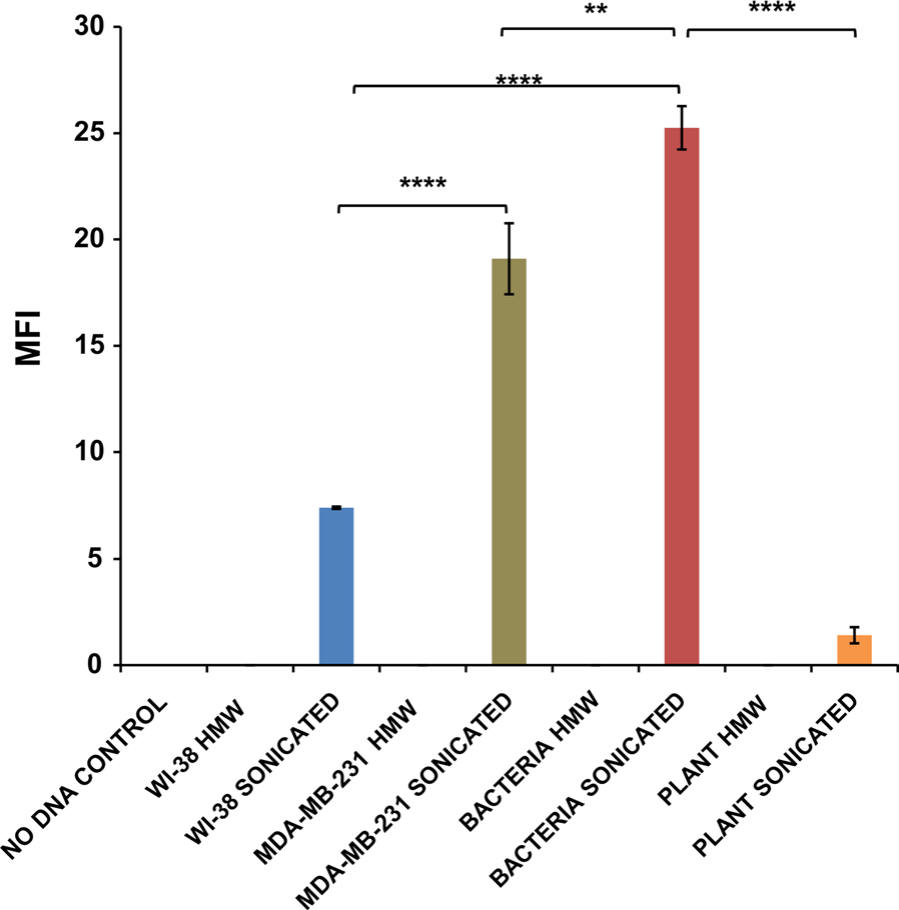
Cellular entry and nuclear uptake of fluorescently labeled HMW and sDNA from various sources by NIH3T3 cells. Images were taken at 30 min following treatment. Quantitative analysis of nuclear fluorescent intensity. **p = 0.01, ****p = 0.0001. *MFI* mean fluorescence intensity. The *horizontal brackets* atop the *bars* represent comparisons made between different pairs of data

We isolated HMW DNA from normal human lung fibroblasts (WI-38 cells), human breast carcinoma cells (MDA-MB-231), bacteria (*E. coli*) and plant (banana) and sonicated them to generate small fragments. Gel analysis of sDNAs revealed a smear with a fragment size range of ~300 to ~3000 bp. However, some fragments >3000 and <300 bp were presumably also present (Additional file 1: Figure S1). This fragment size range is similar to that of nucleic acids isolated from human plasma reported by us and others [4, 5, 16]. We have also shown that these cfDNA fragments are biologically active in doses of 5–10 ng in cell culture assays in 35 mm dishes [4]. We first examined if fluorescently labeled HMW and sDNAs (10 ng each) could access mouse fibroblast cells, especially their nuclei. HMW DNA from none of the above sources showed any intracellular fluorescent signals when examined by laser scanning confocal microscopy (LSCM) (Fig. 1). On the other hand, fluorescent signals could be clearly detected both in cytoplasm and nuclei of mouse cells after treatment with sDNA from all four sources by 30 min (Fig. 1). Bacterial sDNA was taken up by nuclei most avidly while plant sDNA was the least efficient (p = 0.0001) (Fig. 2 and Additional file 2). Although many unknown factors are likely to be involved, variations in methylation patterns between mammalian, bacteria and plant DNA might be an important factor responsible for differential DNA uptake [17, 18].

**Fig. 3:**
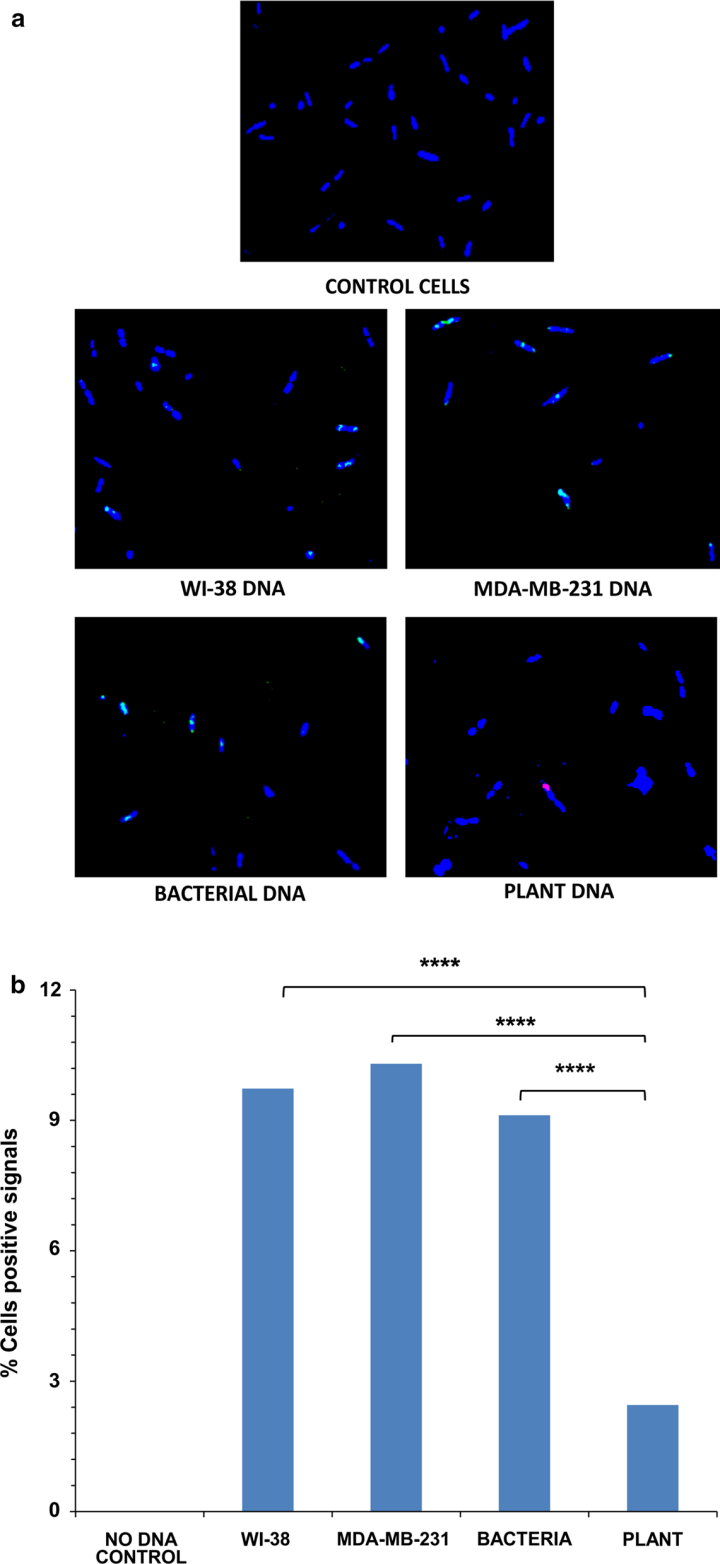
Uptake of various fluorescently labeled sDNAs by bacteria and association with its DNA. **a** Representative fluorescent microscopy images. WI-38, MDA-MB-231 and bacterial DNA are pseudo-coloured as *green*. **b** Quantitative analysis of percentage of cells showing fluorescent signals. ****p = 0.0001. The *horizontal brackets* atop the *bars* represent comparisons made between different pairs of data

Since DNA uptake by bacteria is well documented, we examined if bacteria could take up DNA from non-bacteria sources [19, 20]. When examined by fluorescent microscopy, bacterial cells were found to have taken up sDNAs from mammalian (WI-38 and MDA-MB-231), bacterial (self) and plant sources which were seen to be associated with their DNA by 30 min (Fig. 3a). Approximately 10% of cells showed intracellular signals in case of the two mammalian and bacterial DNA while 2% of the bacteria showed positive signals when treated with plant DNA (Fig. 3b and Additional file 2). HMW DNA were not taken up by bacteria (data not shown). The transformation rate (10%) in case of mammalian cells may seem high; this could be due to the experimental conditions used in the current study.

**Fig. 4:**
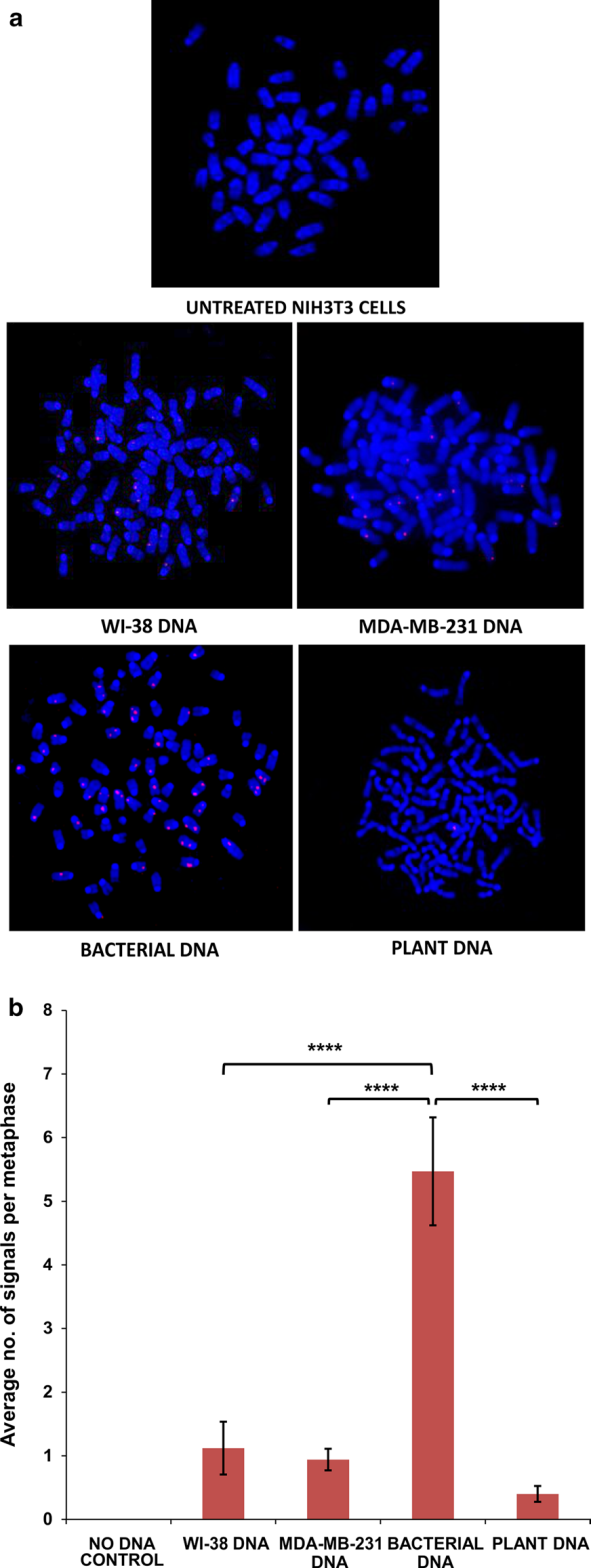
Chromosomal association of sDNA. **a** Representative fluorescent images of metaphase spreads prepared from NIH3T3 cells showing chromosomal association of sDNAs from various sources; **b** average number of fluorescent signals detected per metaphase. ****p = 0.0001. The *horizontal brackets* atop the *bars* represent comparisons made between different pairs of data

**Fig. 5:**
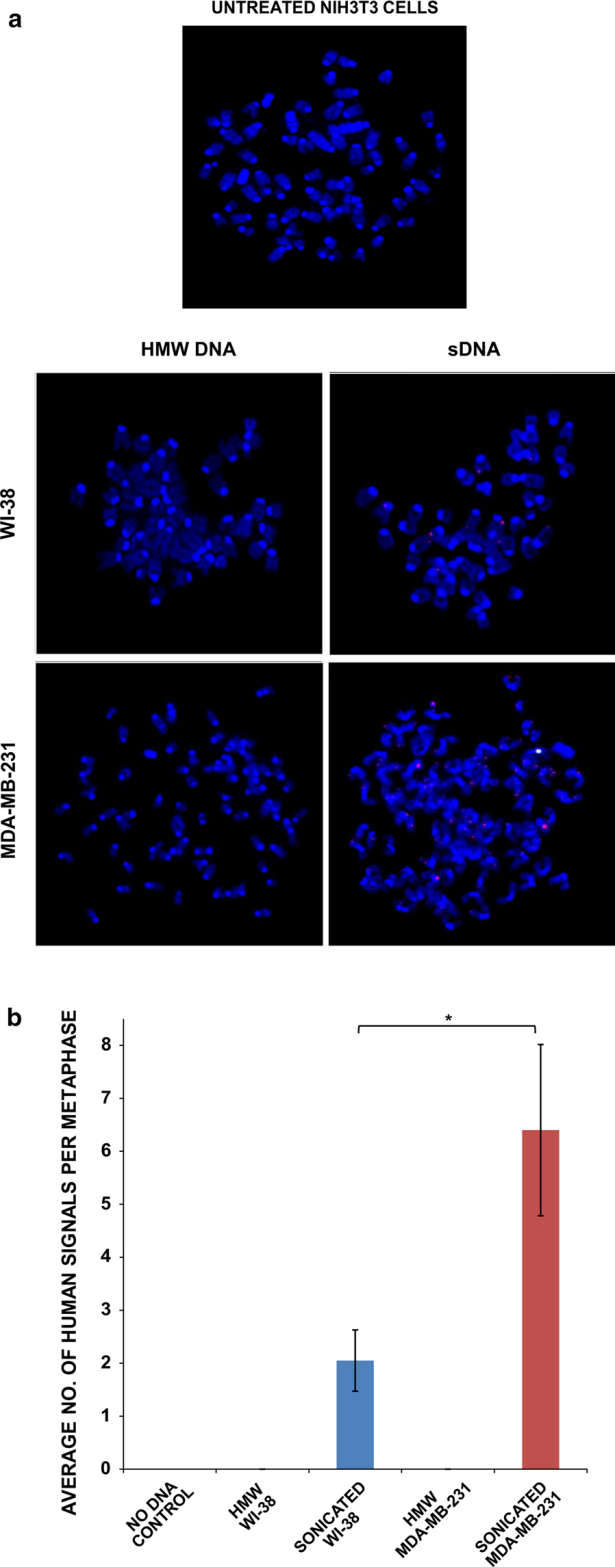
Genomic integration of sDNA. **a** Detection of human cancerous and non-cancerous DNA on chromosomal arms of mouse cells by FISH. *Red signals* represent human genomic DNA; *green signals* represent human pan-centromeric DNA. **b** Quantification of average number of human signals detected per metaphase (n = 50). *p = 0.05. The *horizontal brackets* atop the *bars* represent comparisons made between different pairs of data

We observed that by 30 min, the ingested intra-nuclear sDNAs from all four sources had associated themselves with mouse cell chromosomes (Fig. 4a). Once again, the average number of fluorescent signals per metaphase was the highest in case of bacterial DNA and lowest in case of plant DNA (p = 0.0001) (Fig. 4b and Additional file 2). FISH analysis of metaphase preparations from mouse cells that had been treated with HMW and sDNA from the two human sources at 10th passage showed several positive signals indicating that sDNA had stably integrated into the host cell chromosomes (Fig. 5a). It is likely that incorporation of only larger sonicated fragments were detected by FISH, with smaller fragments escaping detection. Interestingly, significantly more signals were detected per metaphase with respect to sDNA from cancerous human cells (MDA-MB231) than that from normal human cells (WI-38) (Fig. 5b and Additional file 2). This finding is keeping with our earlier report that circulating nucleic acids isolated from cancer patients are biologically more active than those isolated from healthy volunteers [4]. No signals were seen in metaphases from HMW DNA treated cells (Fig. 5b and Additional file 2).

**Fig. 6:**
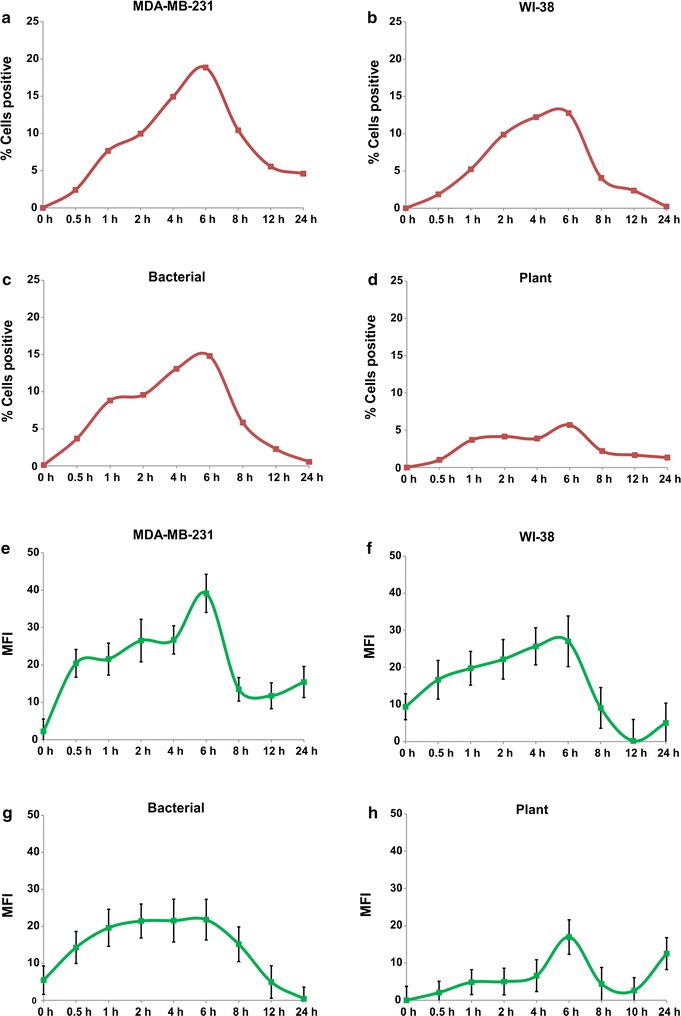
Time course analysis of activation of H2AX (**a**–**d**) and NFκB (**e**–**h**) in NIH3T3 mouse cells in response to sDNA from different sources

**Fig. 7:**
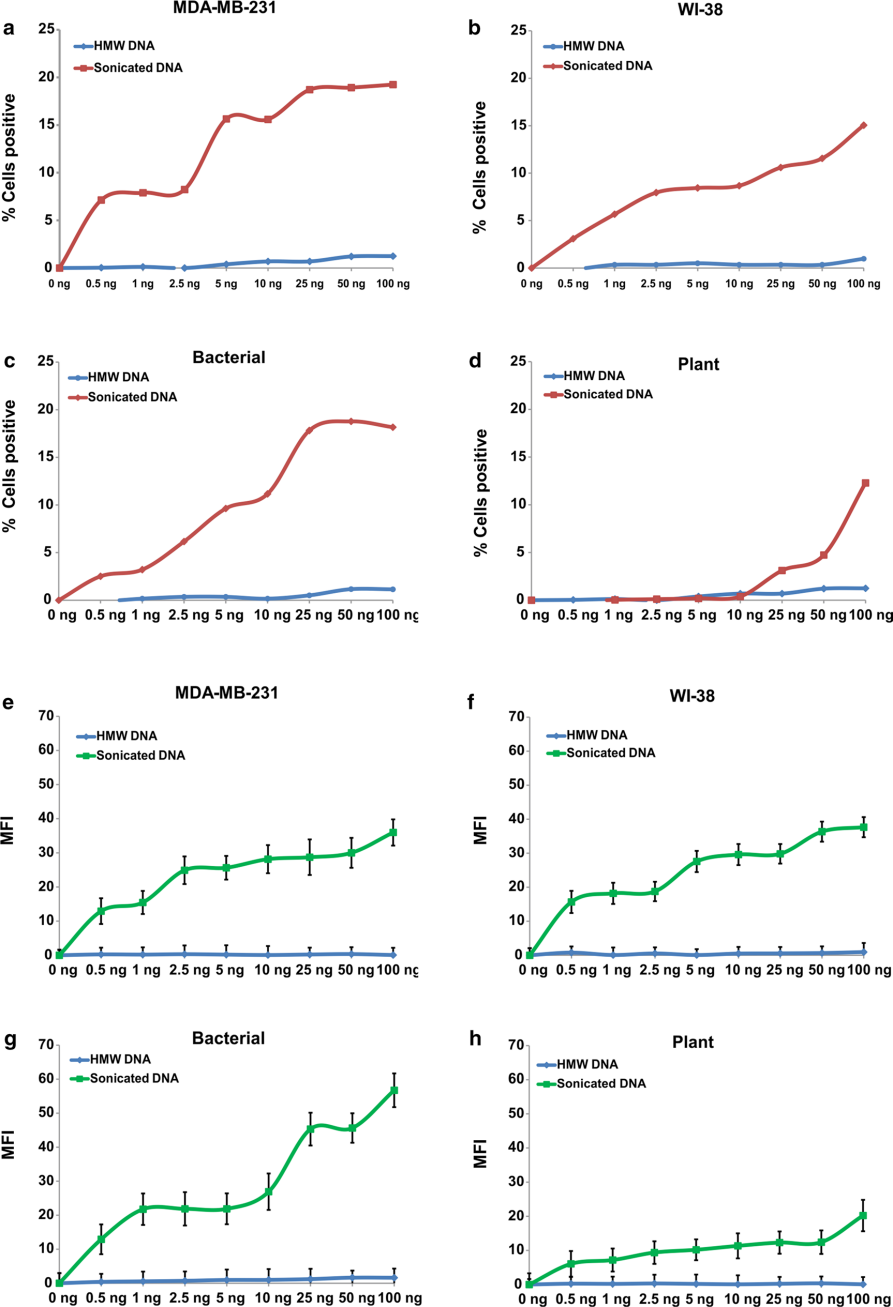
Dose-response analysis of activation of H2AX (**a**–**d**) and NFκB (**e**–**h**) in NIH3T3 mouse cells at 6 h in response to HMW and sDNA from different sources. *MFI* mean fluorescence intensity

We next investigated comparative biological activities of HMW and sDNAs from different sources. The end-points examined were phosphorylation of H2AX and activation of the pro-inflammatory transcription factor NFκB. Treatment of recipient cells with 10 ng of sDNA from all four sources clearly showed up-regulation of both γ-H2AX and NFκB in a time dependent manner that was seen maximally at 6 h with respect to all four sDNAs (Fig. 6a–h and Additional file 2). A dose–response analysis of H2AX and NFκB activation at 6 h showed progressively increasing activation with increasing concentration of sDNA (Fig. 7a–h and Additional file 2). The dose response data indicated that these activities of sDNAs were biological in nature. HMW DNA showed minimal activation of H2AX at high concentrations and little activation of NFκB (Fig. 7a–h and Additional file 2).

Taken together, our results support the hypothesis that cellular/nuclear entry and acquisition of biological activities are functions of size of DNA. These findings are consistent with our earlier observation that fragmented nucleic acids can freely access healthy cells of the body to activate H2AX and active Caspase-3 [4]. The surprising finding in the present report is that, once sonicated, DNA from different species and taxonomical kingdoms could indiscriminately enter into nuclei (or DNA in case of bacteria) of each other without heed to species or kingdom boundaries. Our earlier report that activation of a DDR is necessary for facilitating genomic integration of circulating DNA suggests that a similar DDR activation might be responsible for genomic integration of sDNAs [4].

## Discussion

There is considerable early literature from 1950s and 1960s that suggest that HMW DNA can be taken up by a variety of cells in culture. These have included cells derived from human bone marrow, rat hepatoma, human cervical cancer, mouse embryo and human promyelocytic leukemia, amongst others [21–23]. The internalized DNA reportedly integrated into host cell genomes and could be transcribed and translated into proteins [24]. Chromosomal damage and karyotype alterations were also reported [25]. However, we failed to demonstrate spontaneous uptake of HMW DNA by any of the recipient cells in the present study. This leads us to hypothesize that at least in some of these earlier experiments the HMW DNA might have contained varying quantities of degraded DNA.

The present study was undertaken to investigate our surprising observation that cfDNA derived from dying cells that circulates in human blood are biologically active molecules and can be readily taken up by healthy cells to integrate into their genomes and activate DNA damage and apoptotic responses in the recipients [3, 4, 6]. Since DNA is not conventionally known to be taken up by cells, we hypothesized that ability of DNA to enter into cells is a function of the size of DNA [8, 15]. In order to test this hypothesis, we generated small DNA fragments by sonicating HMW DNA to mimic cell-free DNA that circulates in blood. We have shown here that sDNA from different sources can be indiscriminately taken up by other cells without heed to species or kingdom boundaries. We also show that the uptaken sDNA accumulate in the nuclei of host cells which is followed by their integration into host cell chromosomes. As we have earlier reported that most of the uptaken intra-cellular fragmented cfDNA isolated from plasma are rapidly degraded, the same presumably applies to the uptaken sDNA, with a small fraction that are integrated into host cell chromosomes escaping degradation [4].

Further research is clearly required to address several unanswered questions that are raised by our findings. For example, it remains to be seen if enzymatic digestion of DNA would produce similar results as sonication. Furthermore, the mechanism(s) by which sDNA are taken up by cells is not explained by our study. We observed differences in rates of uptake of sDNA by different cells. For example, sDNAs derived from MDA-MB-231 human cancer cells were taken up more avidly (Figs. 1, 2) and integrated to mouse chromosomes more efficiently than sDNAs from WI-38 normal human fibroblasts (Fig. 5a). Although, this finding is in accordance with our earlier report that circulating DNA fragments isolated from plasma of cancer patients are more active than those from healthy volunteers [4], the reason underlying this differential uptake/activity remains obscure at present. Although the size of cfDNA derived from cancer patients have been reported to be smaller than those from healthy individuals, this cannot explain the differential uptake observed by us, since sonication of HMW DNA from cancerous and non-cancerous sources produced similar sized sDNA (Additional file 1: Figure S1) [26]. It is possible that differences in methylation pattern between normal and cancerous DNA may be responsible for this differential uptake [27]. Our observation that bacterial sDNA was taken up most avidly and associated with chromosomes most efficiently while plant sDNA was least efficient begs for a mechanistic explanation. It should be noted that plant DNA, unlike mammalian and bacterial DNA, was labeled in vitro; however, differential labeling is unlikely to affect rate of uptake of DNA. The possibility that the DNA label itself was being taken up is excluded by our chromosomal association experiments wherein we show discrete, rather than diffuse, fluorescent signals on chromosomes (Fig. 4a). The mechanism by which sDNAs get integrated into host cells genomes will also require further research.

A recent report by Overballe-Petersen et al. [28] is relevant to our study. These authors showed that degraded DNA isolated from 43,000-year old woolly mammoth bone could be taken up by naturally competent environmental bacteria to incorporate small fragments (>20 bp) into their genomes [28]. These transformations involved DNA recombination which was independent of RecA recombinase. The authors suggested that such a natural genetic exchange may have a strong possibility of driving bacterial evolution [28].

We have proposed, based on our earlier findings, that cfDNA that circulate in blood may act as a new class of intra-corporeal mobile genetic elements in light of their ability to enter into healthy cells, integrate into their genomes and activate biological responses [3]. Our present findings extend this possibility by showing that fragmented DNA, irrespective of their source, can be indiscriminately taken up by other cells without heed to species or kingdom boundaries. This leads us to propose that fragmented DNAs may act as mobile genetic elements with evolutionary implications and be involved in horizontal gene transfer (HGT). HGT is known to occur in bacteria and unicellular eukaryotes and thought to play an important role in their evolution [29–31]. Whether HGT occurs in higher organisms is less clear [32–34]. A recent study has, however, provided evidence that for HGT in vertebrate and invertebrate genomes has also occurred in a large scale [35]. However, the mechanism(s) that underlie the transfer of genes between organisms or animals has remained unclear. Our study may help to provide a mechanistic explanation of HGT by suggesting that small fragments of DNA from unicellular organisms and cells of higher species and taxa can be horizontally transferred to others. Our results also suggest a possible mechanism for vertical transfer of genes whereby fragmented extraneous DNA could get incorporated into germ line cells.

Apoptosis, or apoptosis-like processes, are an evolutionarily conserved and are known to occur in bacteria [36, 37], metazoa [38] as well as in higher organisms [39]. Environmental stress is thought to shape adaptation and evolution of species and the role of extreme environmental stress have been emphasized by many [40–42]. We propose that extreme environment stress leading to organismal cell-death and DNA fragmentation/degradation may have played an important role in HGT. The incoming DNA fragments may cause mutations, activate resident genes or even lead to expression of novel genes if the DNA fragments were to be large enough to accommodate such genes.

## Conclusion

We show that while HMW DNAs from mammalian (cancerous and non-cancerous), bacteria and plant sources were incapable of entering into cells, sDNA from the above sources could do so indiscriminately without heed to species or kingdom boundaries. Thus, sDNA from human cells and those from bacteria and plant could enter into nuclei of mouse cells and sDNA from human, bacterial and plant sources could spontaneously enter into bacteria. The intracellular sDNA associated themselves with host cell chromosomes and integrated into their genomes. Furthermore, sDNA, but not HMW DNA, from mammalian, bacterial and plant sources could phosphorylate H2AX and activate the pro-inflammatory transcription factor NFκB in mouse cells, indicating that sDNAs had acquired biological activities.

## Additional files

**Additional file 1: Figure S1.** Agarose gel electrophoretic characterization of HMW and sDNA from various sources.

**Additional file 2.** Data sets. Data generated or analyzed in this study.

## Abbreviations

HMW DNA: high molecular weight DNA
sDNA: sonicated DNA
HGT: horizontal gene transfer
DDR: DNA damage repair response
LSCM: laser scanning confocal microscopy
