# Supplementary material for: Physical shearing imparts biological activity to DNA and ability to transmit itself horizontally across species and kingdom boundaries
Source: BMC Mol Biol. 2017 Aug 9;18:21. doi: 10.1186/s12867-017-0098-8 (PMC5550992; doi:10.1186/s12867-017-0098-8)

**Legend to Additional figure**:

***Additional figure S1:***

Agarose gel electrophoretic characterization of HMW and sDNA from various sources.


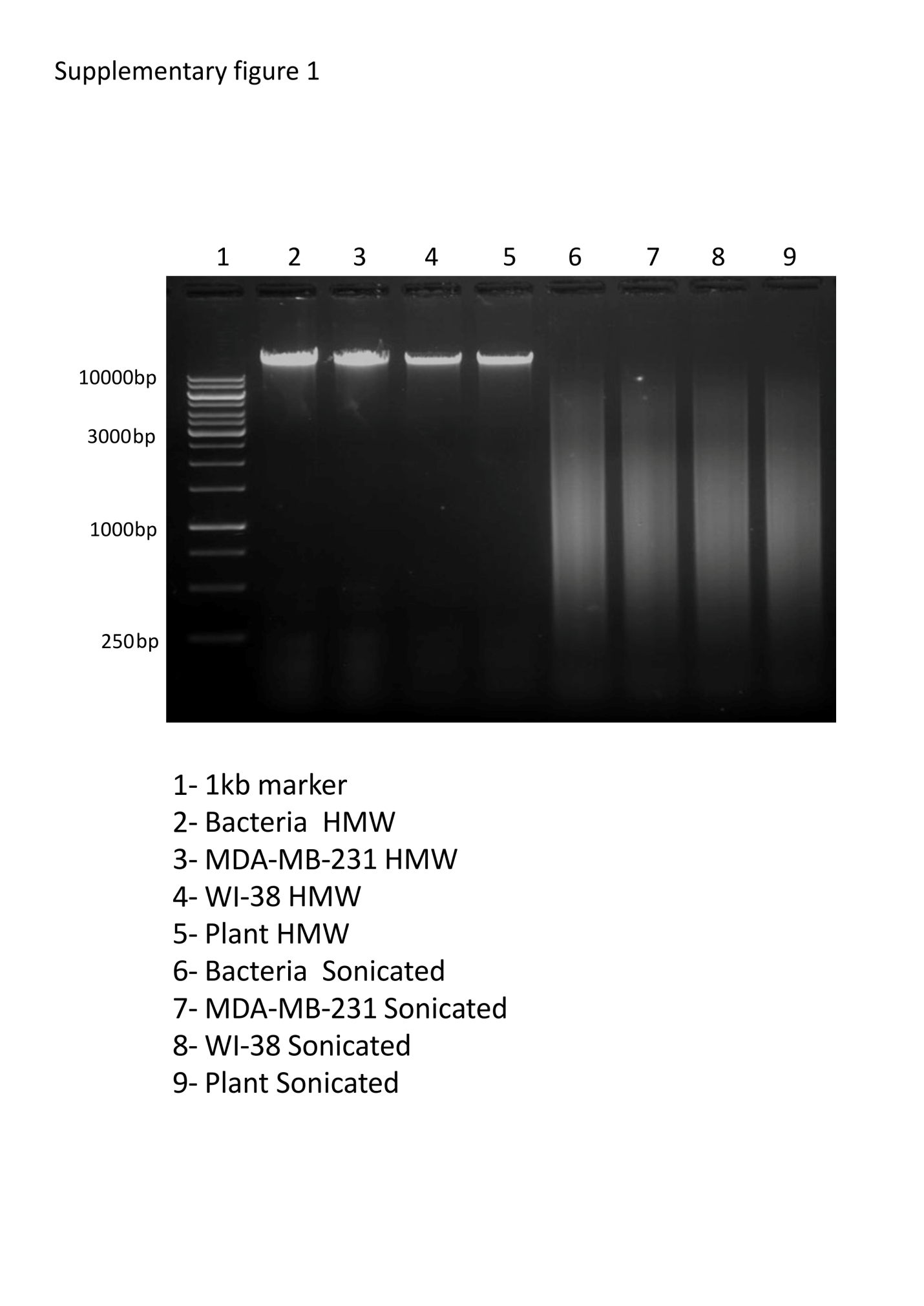

Supplement: Supplementary file 1 — Additional file 1: Figure S1. Agarose gel electrophoretic characterization of HMW and sDNA from various sources. [file 12867_2017_98_MOESM1_ESM.docx]
