# Supplementary material for: Physical shearing imparts biological activity to DNA and ability to transmit itself horizontally across species and kingdom boundaries
Source: BMC Mol Biol. 2017 Aug 9;18:21. doi: 10.1186/s12867-017-0098-8 (PMC5550992; doi:10.1186/s12867-017-0098-8)
Supplement: Supplementary file 2 — Additional file 2. Data sets. Data generated or analyzed in this study. [file 12867_2017_98_MOESM2_ESM.doc]

**Data sets:**

**Data set with respect to Figure 2: Quantitative analysis of nuclear fluorescent intensity**

| **S.No.** | **EXPERIMENT** | **MFI (MEAN ± SEM)** | **P -values** |
| --- | --- | --- | --- |
| a | No DNA Control | 0 ± 0 |  |
| b | WI-38 HMW DNA | 0 ± 0 |  |
| c | WI-38 Sonicated DNA | 7.40 ± 0.05 | c vs g ****p≤0.0001; c vs e ****p≤0.0001 |
| d | MDMAB231-HMW DNA | 0 ± 0 |  |
| e | MDAMB-231 Sonicated DNA | 19.09 ± 1.66 | e vs g **p<0.01 |
| f | Bacterial HMW DNA | 0 ± 0 |  |
| g | Bacterial Sonicated DNA | 25.24 ± 1.01 |  |
| h | Plant HMW DNA | 0 ± 0 |  |
| i | Plant Sonicated DNA | 1.56 ± 0.35 | g vs i ****p≤0.0001 |

**Data set with respect to Figure 3b: Quantitative analysis of percentage of cells showing fluorescent signals**

| **S.No.** | **EXPERIMENT** | **% Cells positive signals (Mean)** | **P -values** |
| --- | --- | --- | --- |
| a | No DNA Control | 0 |  |
| b | WI-38 Sonicated DNA | 9.72973 | b vs e ****p≤0.0001 |
| c | MDAMB-231 Sonicated DNA | 10.30195 | c vs e ****p≤0.0001 |
| d | Bacterial Sonicated DNA | 9.12 | d vs e ****p≤0.0001 |
| e | Plant Sonicated DNA | 2.45283 |  |

**Data set with respect to Figure 4b:** **Chromosomal association of sDNA**

| **S.No.** | **EXPERIMENT** | **Average number of fluorescent signals detected per metaphase**  **(MEAN ± SEM)** | **P -values** |
| --- | --- | --- | --- |
| a | No DNA Control | 0 ± 0 |  |
| b | WI-38 Sonicated DNA | 1.12 ± 0.41 | b vs d ****p≤0.0001 |
| c | MDAMB-231 Sonicated DNA | 0.94 ± 0.17 | c vs d ****p≤0.0001 |
| d | Bacterial Sonicated DNA | 5.47 ± 0.85 |  |
| e | Plant Sonicated DNA | 0.4 ± 0.12 | e vs d ****p≤0.0001 |

**Data set with respect to Figure 5b:** **Genomic integration of sDNA**

| **S.No.** | **EXPERIMENT** | **Average number of human signals detected per metaphase**  **(MEAN ± SEM)** | **P -values** |
| --- | --- | --- | --- |
| a | No DNA Control | 0 |  |
| b | WI-38 HMW DNA | 0 |  |
| c | WI-38 Sonicated DNA | 2.05 | c vs d ****p≤0.0001 |
| d | MDA-MB231-HMW DNA | 0 |  |
| e | MDAMB-231 Sonicated DNA | 6.4 |  |

**Data set with respect to Figure 6** **(a-d): Time course for activation of Gamma H2AX**

| **S.No.** | **EXPERIMENT** | **% cells Positive for Gamma H2AX**  **(MEAN)** | | | | | | | | |
| --- | --- | --- | --- | --- | --- | --- | --- | --- | --- | --- |
|  |  | **0 h** | **0.5 h** | **1 h** | **2 h** | **4 h** | **6 h** | **8 h** | **12 h** | **24 h** |
|  | MDAMB-231 Sonicated DNA | 0 | 2.42 | 7.66 | 9.97 | 14.93 | 18.85 | 10.43 | 5.56 | 4.60 |
|  | WI-38 Sonicated DNA | 0 | 1.87 | 5.235 | 9.88 | 12.23 | 12.76 | 4.07 | 2.37 | 0.23 |
|  | Bacterial Sonicated DNA | 0.11 | 3.67 | 8.80 | 9.56 | 13.07 | 14.80 | 5.83 | 2.26 | 0.53 |
|  | Plant Sonicated DNA | 0 | 1.03 | 3.71 | 4.16 | 3.90 | 5.71 | 2.21 | 1.21 | 1.25 |

**(e-h): Time course for activation of NF-кB**

| **S.No.** | **EXPERIMENT** | **% cells Positive for Gamma H2AX**  **(MEAN ± SEM)** | | | | | | | | |
| --- | --- | --- | --- | --- | --- | --- | --- | --- | --- | --- |
|  |  | **0 h** | **0.5 h** | **1 h** | **2 h** | **4 h** | **6 h** | **8 h** | **12 h** | **24 h** |
|  | MDAMB-231 Sonicated DNA | 2.24± 3.30 | 20.42 ± 3.71 | 21.56 ± 4.27 | 26.51 ± 5.69 | 26.70 ± 3.77 | 39.14 ± 5.10 | 13.48 ± 3.14 | 11.76 ± 3.45 | 15.43 ± 4.17 |
|  | WI-38 Sonicated DNA | 9.39  ±  3.50 | 16.66  ±  5.20 | 19.76  ±  4.55 | 22.18  ±  5.32 | 25.67  ±  5.00 | 27.03  ± 6.82 | 9.08  ±  5.49 | 0.19  ±  5.75 | 5.01  ±  5.35 |
|  | Bacterial Sonicated DNA | 5.46  ±3.82 | 14.30  ± 4.32 | 19.61  ± 5.00 | 21.45  ± 4.58 | 21.55  ± 5.78 | 21.80  ± 5.49 | 15.16  ± 4.68 | 4.96  ± 4.37 | 0.42  ± 3.15 |
|  | Plant Sonicated DNA | 0 ±3.74 | 2.07  ±  3.02 | 4.86  ±  3.33 | 5.02  ±  3.57 | 6.61  ±  4.26 | 16.96  ±  4.63 | 4.39  ±  4.41 | 2.62  ±  3.45 | 12.52  ±  4.27 |

**Data set with respect to Figure 7** **(a-d): Dose-response analysis of activation of H2AX in NIH3T3 mouse cells at 6 hr in response to HMW and sDNA from different sources**

| **S.No.** | **EXPERIMENT** | **MFI for Gamma H2AX**  **(MEAN)** | | | | | | | | |
| --- | --- | --- | --- | --- | --- | --- | --- | --- | --- | --- |
|  |  | **0 ng** | **0.5 ng** | **1 ng** | **2.5 ng** | **5 ng** | **10 ng** | **25 ng** | **50 ng** | **100 ng** |
| 1. c | MDMAB231-HMW DNA | 0 | 0.04 | 0.12 | 0 | 0.39 | 0.69 | 0.69 | 1.21 | 1.25 |
| 1. e | MDAMB-231 Sonicated DNA | 0 | 7.13 | 7.89 | 8.24 | 15.64 | 15.59 | 18.71 | 18.93 | 19.24 |
| 1. g | WI-38 HMW DNA | 0 | -0.11 | 0.34 | 0.34 | 0.50 | 0.34 | 0.340 | 0.340 | 0.98 |
|  | WI-38 Sonicated DNA | 0 | 3.09 | 5.67 | 7.95 | 8.44 | 8.67 | 10.60 | 11.56 | 15.05 |
|  | Bacterial HMW DNA | 0 | -0.10 | 0.16 | 0.35 | 0.35 | 0.16 | 0.50 | 1.16 | 1.14 |
|  | Bacterial Sonicated DNA | 0 | 2.51 | 3.21 | 6.16 | 9.63 | 11.16 | 17.81 | 18.77 | 18.16 |
|  | Plant HMW DNA | 0 | 0.04 | 0.12 | 0 | 0.39 | 0.69 | 0.69 | 1.21 | 1.25 |
|  | Plant Sonicated DNA | 0 | -0.14 | 0.03 | 0.11 | 0.19 | 0.39 | 3.12 | 4.74 | 12.29 |

**(e-h): Dose-response analysis of activation of NFкB in NIH3T3 mouse cells at 6 hr in response to HMW and sDNA from different sources**

| **S.No.** | **EXPERIMENT** | **MFI for NF-кB**  **(MEAN ± SEM)** | | | | | | | | |
| --- | --- | --- | --- | --- | --- | --- | --- | --- | --- | --- |
|  |  | **0 ng** | **0.5 ng** | **1 ng** | **2.5 ng** | **5 ng** | **10 ng** | **25 ng** | **50 ng** | **100 ng** |
|  | MDMAB231-HMW DNA | 0± 1.63 | 0.27 ± 1.97 | 0.20 ± 2.13 | 0.33 ± 2.55 | 0.19 ± 2.75 | 0.09 ± 2.62 | 0.24 ± 2.00 | 0.35 ± 2.02 | 0.09 ± 2.12 |
|  | MDAMB-231 Sonicated DNA | 0 ± 1.63 | 12.94 ± 3.77 | 15.47 ± 3.39 | 24.91 ± 4.05 | 25.64 ± 3.47 | 28.16 ± 4.12 | 28.73 ± 5.21 | 30.02 ± 4.39 | 35.98 ± 3.84 |
|  | WI-38 HMW DNA | 0 ± 2.09 | 0.77  ± 1.81 | 0.08  ± 2.20 | 0.55  ±1.78 | 0.07  ±1.73 | 0.50 ±1.95 | 0.564 ± 1.87 | 0.65 ± 1.96 | 0.93 ± 2.68 |
|  | WI-38 Sonicated DNA | 0 ± 2.09 | 15.68 ± 3.26 | 18.21 ± 3.11 | 18.75± 2.86 | 27.57 ± 3.11 | 29.61 ± 3.100 | 29.83 ± 2.80 | 36.35 ± 2.95 | 37.66 ± 2.96 |
|  | Bacterial HMW DNA | 0 ±2.98 | 0.41 ± 2.34 | 0.56 ± 2.86 | 0.70 ± 2.76 | 0.96 ± 3.06 | 0.98 ± 3.19 | 1.22 ± 3.02 | 1.64 ± 2.08 | 1.63 ± 2.68 |
|  | Bacterial Sonicated DNA | 0 ± 2 .98 | 12.92 ± 4.38 | 21.77 ± 4.63 | 21.88 ± 4.90 | 21.89 ± 4.54 | 26.92 ± 5.36 | 45.32 ± 4.82 | 45.65 ± 4.33 | 56.74 ± 4.96 |
|  | Plant HMW DNA | 0.00 ± 1.63 | 0.27±1.97 | 0.20±2.13 | 0.33±2.55 | 0.19±2.75 | 0.09±2.62 | 0.24±2.00 | 0.35±2.02 | 0.09±2.12 |
|  | Plant Sonicated DNA | 0.00 ± 3.30 | 6.08 ± 3.75 | 7.19± 3.37 | 9.37 ±3.31 | 10.19 ± 3.06 | 11.35 ± 3.67 | 12.30 ± 3.26 | 12.42 ± 3.45 | 20.22 ± 4.60 |
